# CK2 inhibition with silmitasertib promotes methuosis-like cell death associated to catastrophic massive vacuolization of colorectal cancer cells

**DOI:** 10.1038/s41419-019-1306-x

**Published:** 2019-01-25

**Authors:** Eduardo Silva-Pavez, Paulina Villar, César Trigo, Esteban Caamaño, Ignacio Niechi, Pablo Pérez, Juan P. Muñoz, Francisco Aguayo, Verónica A. Burzio, Manuel Varas-Godoy, Ariel F. Castro, María I. Colombo, Julio C. Tapia

**Affiliations:** 1 Departamento de Oncología Básico Clínica, Facultad de Medicina, Universidad de Chile, Santiago, Chile; 2 Fundación Ciencia & Vida, Andes Biotechnologies SpA, Facultad de Ciencias de la Vida, Universidad Andrés Bello, Santiago, Chile; 3 Centro de Investigación Biomédica, Facultad de Medicina, Universidad de Los Andes, Santiago, Chile; 4 Departamento de Bioquímica y Biología Molecular, Facultad de Ciencias Biológicas, Laboratorio de Transducción de Señales y Cáncer, Universidad de Concepción, Concepción, Chile; 5 Laboratorio de Biología Celular y Molecular, Instituto de Histología y Embriología, Facultad de Ciencias Médicas, CONICET, Universidad Nacional de Cuyo, Mendoza, Argentina; 6 Present Address: Laboratorio de Patología Molecular, Instituto de Bioquímica y Microbiología, Facultad de Ciencias, Universidad Austral de Chile, Valdivia, Chile

## Abstract

Protein kinase CK2 is a highly conserved and constitutively active Ser/Thr-kinase that phosphorylates a large number of substrates, resulting in increased cell proliferation and survival. A known target of CK2 is Akt, a player in the PI3K/Akt/mTORC1 signaling pathway, which is aberrantly activated in 32% of colorectal cancer (CRC) patients. On the other hand, mTORC1 plays an important role in the regulation of protein synthesis, cell growth, and autophagy. Some studies suggest that CK2 regulates mTORC1 in several cancers. The most recently developed CK2 inhibitor, silmitasertib (formerly CX-4945), has been tested in phase I/II trials for cholangiocarcinoma and multiple myeloma. This drug has been shown to induce autophagy and enhance apoptosis in pancreatic cancer cells and to promote apoptosis in non-small cell lung cancer cells. Nevertheless, it has not been tested in studies for CRC patients. We show in this work that inhibition of CK2 with silmitasertib decreases in vitro tumorigenesis of CRC cells in response to G2/M arrest, which correlates with mTORC1 inhibition and formation of large cytoplasmic vacuoles. Notably, molecular markers indicate that these vacuoles derive from massive macropinocytosis. Altogether, these findings suggest that an aberrantly elevated expression/activity of CK2 may play a key role in CRC, promoting cell viability and proliferation in untreated cells, however, its inhibition with silmitasertib promotes methuosis-like cell death associated to massive catastrophic vacuolization, accounting for decreased tumorigenicity at later times. These characteristics of silmitasertib support a potential therapeutic use in CRC patients and probably other CK2-dependent cancers.

## Introduction

Colorectal cancer (CRC) is a multifactorial disease affecting millions of people worldwide and has been linked to deregulation of several signaling pathways. The PI3K/Akt signaling pathway plays an important role in a variety of cancers due to its association with processes that promote proliferation, resistance to apoptosis, invasion, and metastasis^1^. In CRC, a number of genetic and epigenetic alterations have been described, for example, activating mutations in the PI3K kinase gene have been identified in 32% of tumors^2^, as well as loss of function mutations of the tumor suppressor PTEN^3^. All these alterations contribute to the aberrant activation of the PI3K/Akt signaling pathway and, in consequence, acquisition of a metastatic phenotype^4^.

A key downstream component of the PI3K/Akt signaling pathway is the mammalian target of rapamycin complex 1 (mTORC1), which plays an important role in different types of cancer, including CRC^4,5^. The core component of this complex, the mammalian target of rapamycin (mTOR), is a highly conserved Ser/Thr-kinase that integrates growth factor and nutritional signals to promote growth and survival of normal cells. Activation of mTORC1 leads to phosphorylation of mediators of protein translation and cell growth, including the ribosomal S6 kinase 1 (S6K1) and 4EBP1^6,7^. MTORC1 plays an important role in the regulation of protein synthesis, cell growth and autophagy in response to nutrients and growth factors^8^. Inactivation of TSC2 by Akt favors the activation of Rheb, which interacts and activates mTORC1 at the lysosomal membrane^8,9^. Inhibition mTORC1 was shown to decrease formation of polyps, oncogenesis, and mortality of ApcΔ716 mice^10^. Also, treatment with rapamycin leads to a reduction of tumors in an in vivo model of PI3K-dependent CRC^11^.

Autophagy is initiated by ULK-1, which is activated under nutrient deprivation or mTORC1 inhibition by rapamycin^12–14^. Autophagy is associated to a number of diseases, although its role in tumorigenesis and progression is controversial^12,15^. Some studies show that autophagy suppresses tumorigenesis^15,16^, while in others autophagy inhibition by silencing Rheb decreases survival of Colo320HSR colon cancer cells^17^. Likewise, autophagy inhibition exerts an anticancer effect in HCT-116 colon cancer cells by triggering apoptosis^18^. Conversely, a dual inhibitor of mTORC1/2, WYE354, induces autophagy and activates apoptosis in HCT-116 and HT-29 colon cancer cells^19^. Finally, Beclin-1 overexpression correlates with a positive prognosis and survival of CRC patients^20^.

Protein kinase CK2 has been proposed as a therapeutic target in various cancers. CK2 is a highly conserved constitutively active Ser/Thr-kinase capable of phosphorylating a large number of substrates, increasing proliferation, and survival^21–23^. CK2 is able to control mTORC1 in several cancers. In fact, CK2 regulates the PI3K/Akt pathway through phosphorylation of Akt at Ser-129, causing its hyperactivation^24,25^. Thus, CK2 silencing has been tested and greater effort dedicated to study specific inhibitors for therapy. The latest developed CK2 inhibitor, silmitasertib (formerly CX-4945), displays excellent pharmacological properties, which rendered it suitable for evaluation in phase I/II trials for cholangiocarcinoma and multiple myeloma (clinicaltrials.gov). Despite it has not yet been included in studies for CRC patients, it induces in vitro autophagy and enhances apoptosis in pancreatic cancer cells^26^, as well as promotes apoptosis in non-small cell lung cancer cells by inhibiting the PI3K/Akt/mTOR pathway^27^. In addition, silmitasertib induces apoptosis in epidermoid carcinoma and squamous carcinoma cells by a complete inhibition of the PI3K/Akt/mTOR pathway in combination with erlotinib^28^.

Here, we show that an aberrantly elevated expression/activity of CK2 may play an undescribed role in viability of CRC cells. Thus, CK2 inhibition with silmitasertib promotes an early methuosis-like cell death, which is associated to massive catastrophic macropinocytosis, accounting for abolition of tumorigenicity at later stages. These results support the use of silmitasertib as a promissory therapeutic alternative in the treatment of CRC patients.

## Results

### CK2 inhibition decreases tumorigenesis of CRC cells through a G2/M arrest

**Fig. 1:**
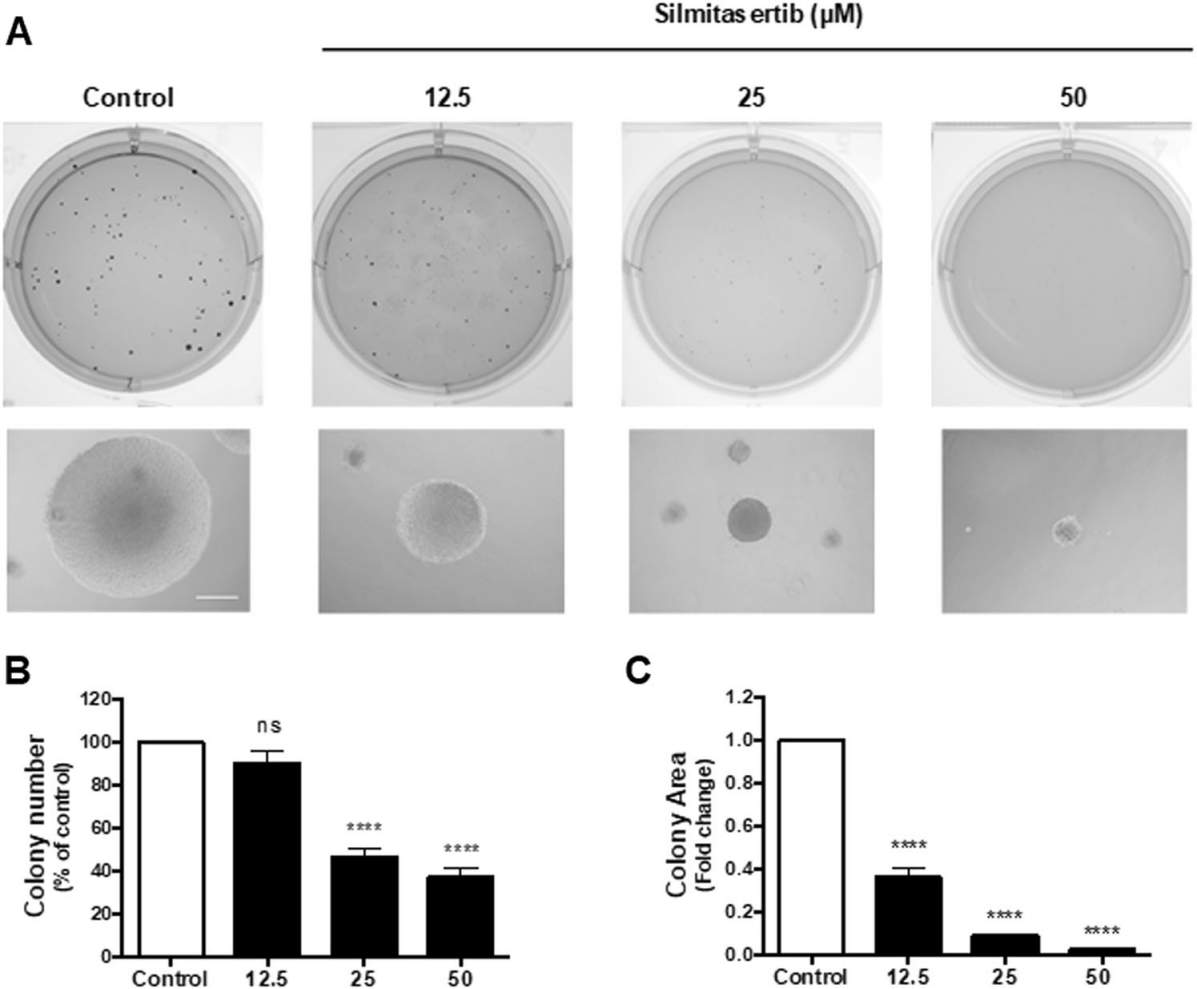
CK2 inhibition with silmitasertib decreases in vitro tumorigenesis of CRC cells. Anchorage-independent colony growth in soft-agar was determined in DLD-1 cells treated with increasing concentrations of silmitasertib for 21 days. **a** Representative images of colonies in soft-agar of control (white bars) and silmitasertib-treated (black bars) cells. The number of colonies per well (**b**) and area of colonies (**c**) was plotted. Data represents the average ± SEM (*n* = 3); ****p < 0.0001; ns, not significant. Bar: 20 μm

**Fig. 2:**
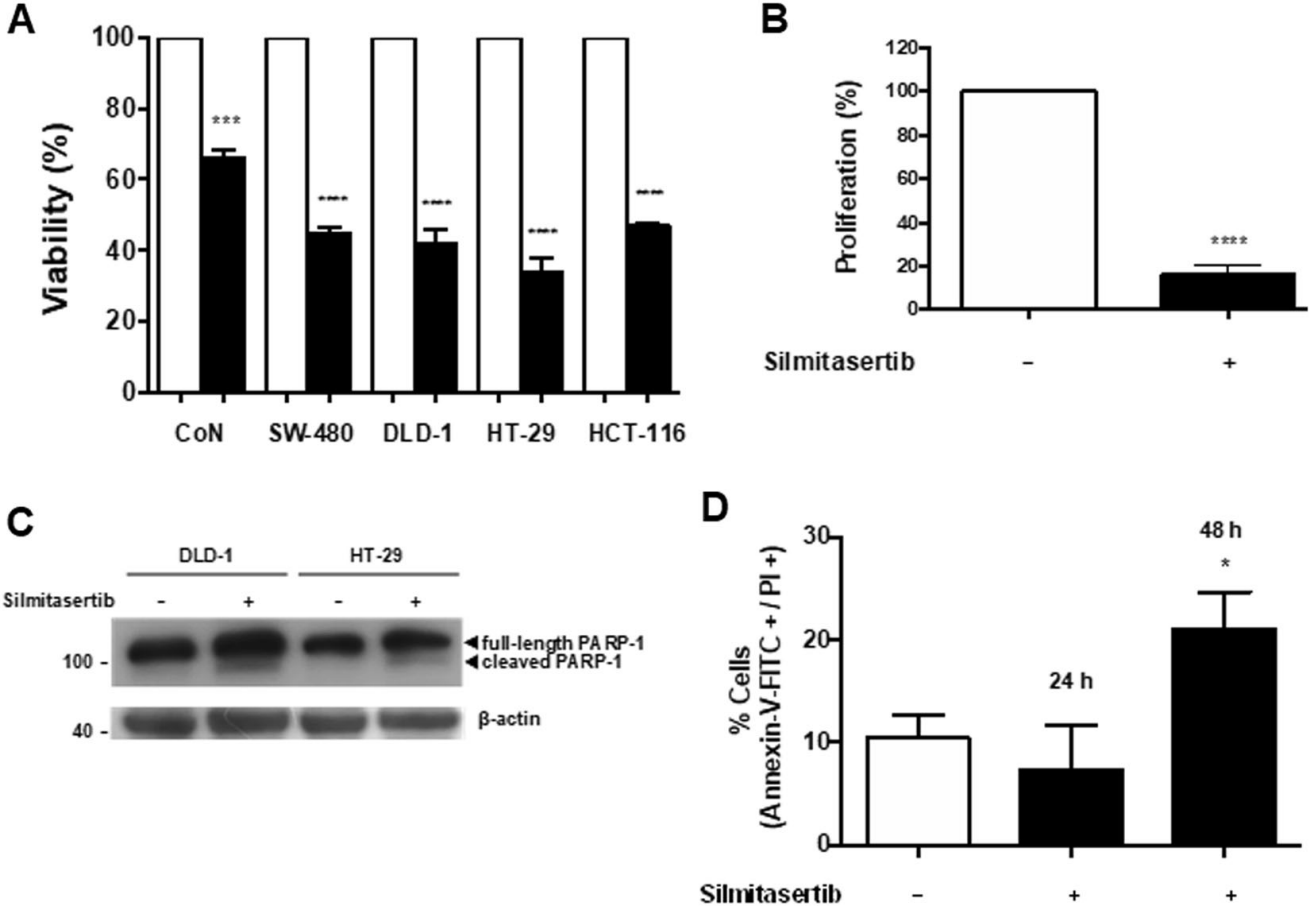
Silmitasertib decreases viability and proliferation of CRC cells. **a** Cell viability was determined by MTS assay in normal (CoN) and CRC cells (SW-480, DLD-1, HT-29, and HCT-116) after incubation with either vehicle (white bars) or 25 μM silmitasertib (black bars) for 48 h. **b** DLD-1 cells were treated as in **a** and proliferation was determined by CFSE staining and cytometry. **c** Protein levels of caspase-cleaved PARP-1 were detected by western blot in DLD-1 and HT-29 cells treated as in **a**. **d** DLD-1 cells treated with 25 μM silmitasertib for 24 and 48 h were analyzed through double positive annexin-V-FITC/PI staining by cytometry. Data represents the average ± SEM (*n* = 3); *p < 0.05

Tumorigenic cells have the in vitro capacity of anchorage-independent growth and forming colonies in soft-agar. Thus, we studied whether CK2 inhibition could decrease clonogenesis of DLD-1 cells after 21 days of treatment with increasing concentrations of silmitasertib. A significant dose-dependent decrease in both number and size of the colonies was observed (Fig. 1), suggesting a potent effect of silmitasertib in decreasing CRC tumorigenesis. To elucidate the underlying mechanism, a tenfold shorter time was used to evaluate whether silmitasertib affects the viability of DLD-1 and other CRC cells of different malignancy^29^. SW-480 (Dukes B state), DLD-1 (Dukes C state), HT-29 (Dukes C/D state), and HCT-116 (Dukes D state) cells were grown with or without 25 μM silmitasertib for 48 h. Viability of all CRC cells decreased 60–70%, while normal colon epithelial CoN cells only decreased just over 30% (Fig. 2a). Decreased viability of CRC cells correlated with diminished proliferation (Fig. 2b) and late apoptosis, as observed by PARP-1 cleavage and annexin-V^+^/PI^+^ staining after 48 h (Fig. 2c, d), however, the pan-caspase inhibitor Z-VAD-FMK was unable to revert the decreased viability both at 24 h and 48 h (Supp Fig. 1A). Since double annexin-V^+^/PI^+^ staining is also related to early necrosis/necroptosis, we assessed whether the decreased viability may probably derive from this at 48 h. The necroptosis inhibitor, necrostatin-1s (Nec1s), did not revert the viability neither at 24 h nor at 48 h (Supp Fig. 1B). Additionally, localization of High Mobility Group Box 1 (HMGB1) transcription factor, which is exported to the cytoplasm during early necrosis^30^, showed only a minimal cytoplasmic localization at 12 h of treatment (Supp Fig. 1C).

**Fig. 3:**
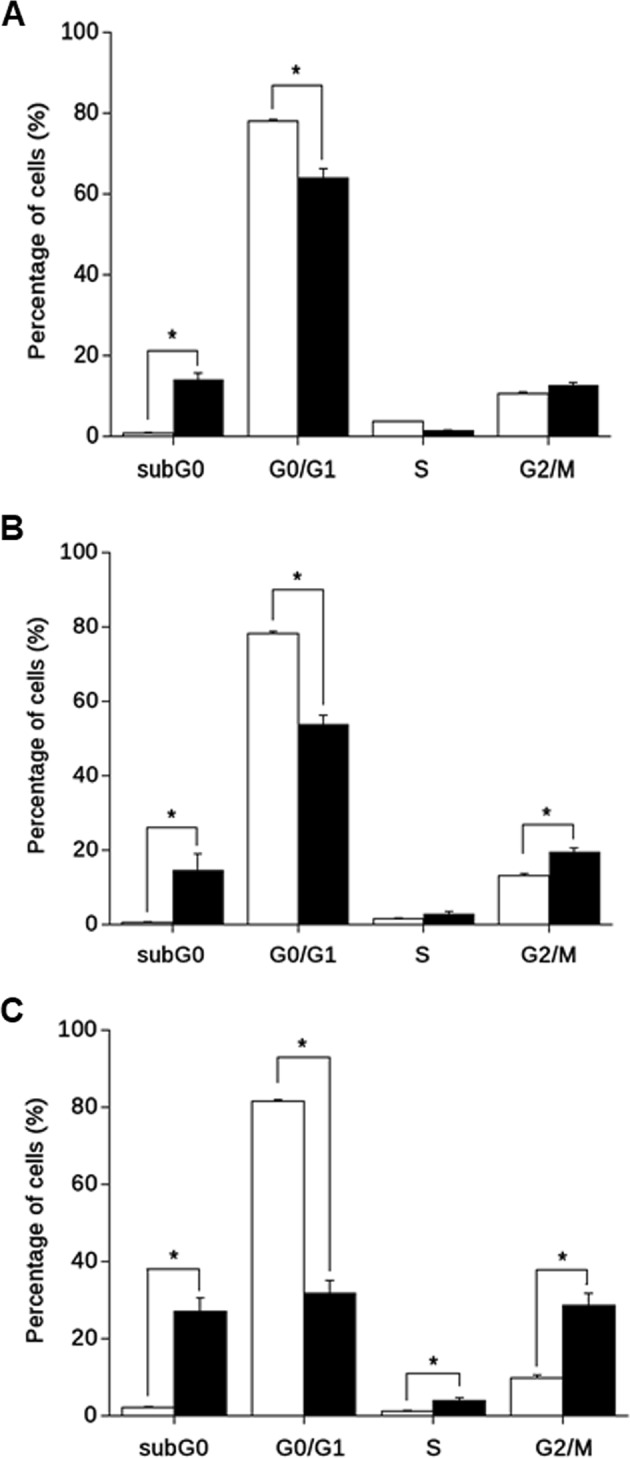
Silmitasertib induces G2/M arrest in CRC cells. Cell cycle distribution was determined by flow cytometry of PI-stained DLD-1 cells untreated (white bars) or treated (black bars) with 25 μM silmitasertib for 16 h (**a**), 24 h (**b**), and 48 h (**c**). The percentage of cells in the different cell cycle phases is plotted. Data represents the average ± SEM (*n* = 3), *p < 0.05

A more specific analysis of silmitasertib treatment on cell cycle was performed. This analysis showed a basal sub-G_0_ fraction at 16 h (Fig. 3a) that was maintained at 24 h (Fig. 3b) but, as expected, significantly progressed at 48 h (Fig. 3c). In parallel, a reduction in the *G*_0_/*G*_1_ fraction was already observed at 16 h, which progressed through 24 h and further at 48 h. Notably, an increase in G2/M arrest was observed at 24 h, which was much more elevated at 48 h of treatment. Activating mutation of the *PIK3CA* gene is a common trait in CRC tumors, leading to aberrant activation of the PI3K/Akt pathway^2^, however, silmitasertib triggered the same G2/M arrest in SW-480 cells (Supp Fig. 2) which, unlike DLD-1, does not contain activating mutations in *PIK3CA*. These results suggest an effect of the CK2 inhibitor silmitasertib on decreasing tumorigenicity of CRC cells through a G2/M arrest.

### CK2 regulates mTORC1 activity in CRC cells

**Fig. 4:**
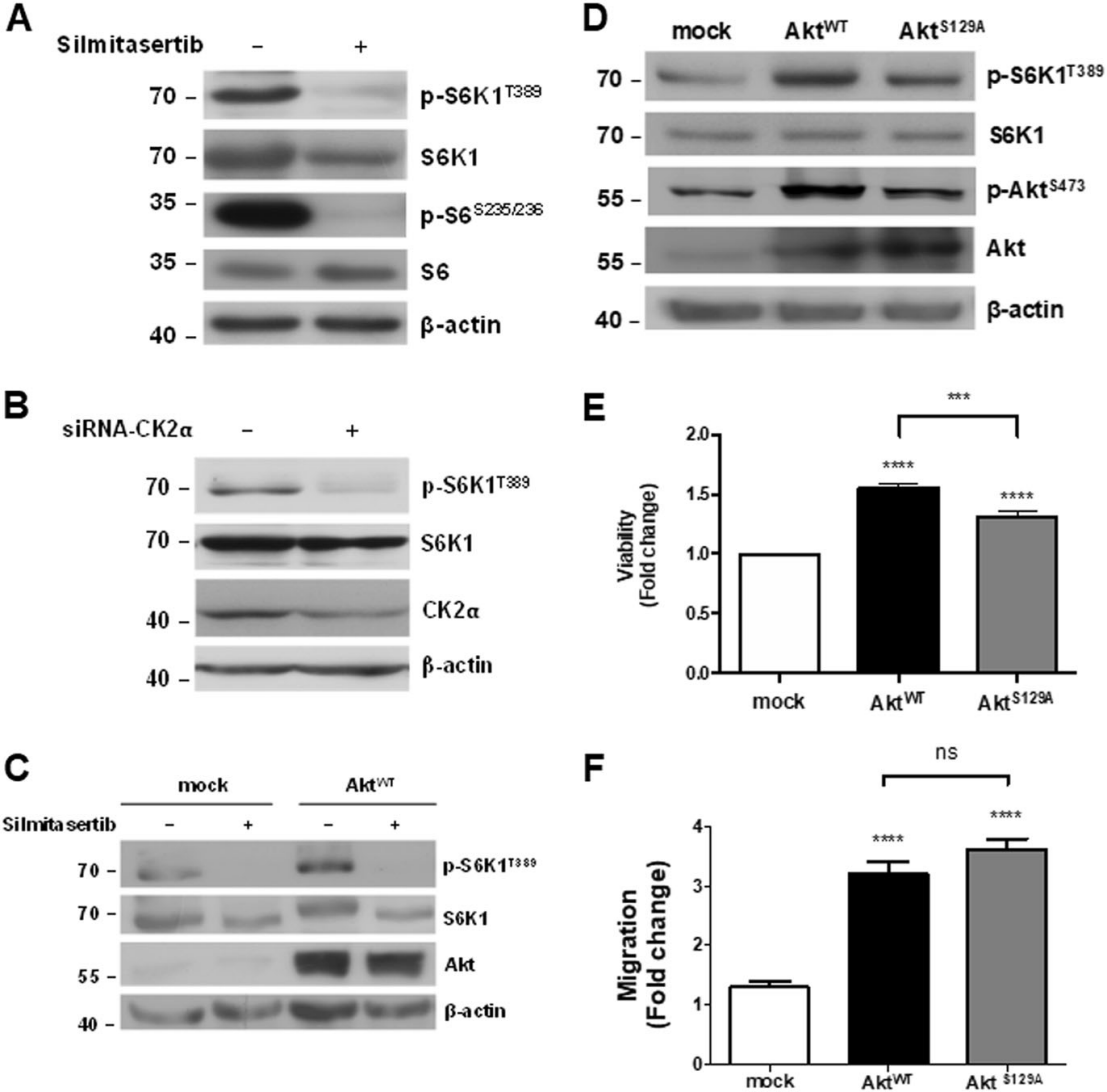
Decreased mTORC1 activity in CRC cells treated with silmitasertib. **a** DLD-1 cells were incubated with 25 μM silmitasertib for 16 h. The levels of the indicated proteins were analyzed by western blot. **b** Cells were transfected with 100 nM of a specific siRNA for CK2α. The indicated proteins were analyzed by western blot. **c** Cells were transfected with the plasmid pCMV6-myr-AKT-1-CA-HA and then treated with 25 μM silmitasertib for 16 h. Protein levels were analyzed by western blot. **d** Levels of indicated proteins in cells transfected with the plasmids pCMV6-myr-AKT-1-CA-HA or pCMV6-myr-AKT-1-S129A-HA were analyzed by western blot. **e** Cell viability determined by MTS assay of cells transfected as in **d**. **f** Cell migration determined by transwell assay of cancer cells transfected as in **d**. Data represents the average ± SEM (*n* = 3); **p < 0.001; ****p < 0.0001; ns: not significant

Since mTORC1 is activated during mitosis and is therefore important in G2/M cell cycle progression, as well as in maintaining genomic integrity during this period^27,31–34^, we assessed whether the G2/M arrest could be a consequence of reduced mTOR activity. Thus, phosphorylations on S6K1 at Thr389 and S6 at Ser235/Ser236 were used as activity markers of kinases mTORC1 and S6K1, respectively. As observed in Fig. 4a, treatment with silmitasertib for 16 h led to reduced activity of both kinases. To confirm that the effect on mTORC1 is specific of CK2, expression of CK2α was decreased with a specific siRNA^35^ and levels of pT389-S6K1 were analyzed. Concordantly, a similar decrease in pT389-S6K1 levels was observed as early as 16 h upon silencing of CK2α (Fig. 4b). Given that mTORC1 is activated by Akt, we evaluated whether Akt overexpression reversed the negative effect of silmitasertib on mTORC1-dependent phosphorylation of S6K1. Overexpression of full-length wild-type Akt (Akt-WT) did not reverse the decrease in mTORC1 activity after inhibition of CK2 (Fig. 4c). CK2 is also described to phosphorylate Akt at Ser-129, which is important for the Wnt/β-catenin pathway in HEK-293 cells^36,37^. We analyzed whether overexpression of a mutant Akt unable to be phosphorylated by CK2 (Akt-S129A) had an effect on mTORC1 activity. The inability of being phosphorylated by CK2 slightly precluded Akt-S129A of activating mTORC1 (Fig. 4d and Supp Fig. 3A), which probably affected its capability to increase viability (Fig. 4e), however, it did not affect the augmented migration of Akt-S129A-expressing cells as compared to Akt-WT cells (Fig. 4f). This suggests that Ser-129 is somehow important for promoting viability of DLD-1 cells by probably promoting a full activation of mTORC1, which is evidenced for a slight decrease of pT389-S6K1. However, this residue would be expendable for the PI3K/Akt-promoted migration.

Nutrients and growth factors, as well as altered PI3K/Akt signaling, have been shown to promote subcellular localization of mTORC1 to the outer layer of lysosomal membranes, where it is activated by Rheb^6,8,9^. We evaluated whether silmitasertib affected the localization of mTORC1 at lysosomes of DLD-1 cells. As early as 6 h, untreated cells showed perinuclear localization of lysosomes with the expected colocalization of mTORC1, while in treated cells lysosomes were more disperse and apparently smaller, and their colocalization with mTORC1 was reduced (Supp Fig. 3B), suggesting that CK2 inhibition hindered localization of mTORC1 to lysosomes and probably its activation by Rheb. MTORC1 is also known to be a key regulator of autophagy^38^. In fact, mTORC1 inhibition has been linked to augmented autophagy in different cancer cell lines^8,19,39^. Lysosomal Rheb-activated mTORC1 inhibits ULK-1 by phosphorylation at Ser757, which is key for early autophagy induction^13,14^. Thus, augmented pSer757-ULK-1 levels are a good marker of autophagy blockage. Therefore, to assess whether CK2 inhibition could have an impact on the activation of ULK-1, cells were treated with silmitasertib for 6 h, following analysis of mTORC1 and ULK-1 phosphorylation by western blot. As expected, silmitasertib led to decreased levels of mTORC1-dependent phosphorylation in both targets, pT389-S6K1 and pS757-ULK-1 (phospho-inhibitory site), thus meaning that mTORC1 and ULK-1 were inhibited and more active (i.e., less-inhibited), respectively, as compared with untreated cells (Supp Fig. 3C). Altogether, these results indicate that CK2 inhibition may lead to inactivation of mTORC1 in CRC cells.

### Silmitasertib-dependent mTORC1 inhibition leads to formation of large vacuoles

**Fig. 5:**
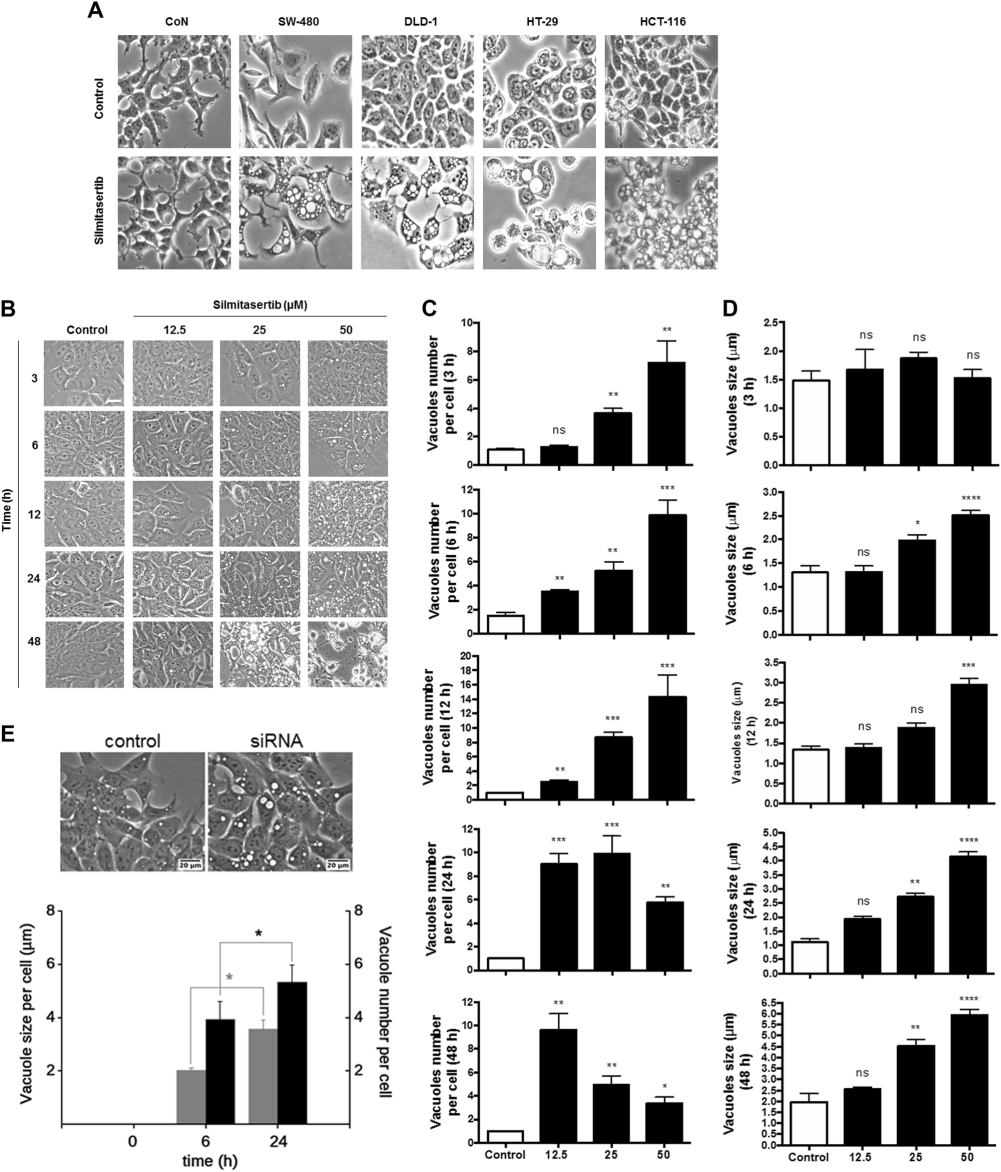
MTORC1 inhibition correlates with ULK-1 activation and large acidic vacuoles. **a** Colon cancer (SW-480, DLD-1, HT-29, and HCT-116) and non-tumor (CoN) cells were treated with 25 μM silmitasertib for 6 h and photographed under a phase-contrast microscope. **b** DLD-1 cells were treated with silmitasertib at increasing concentrations (12.5, 25, and 50 μM) for different times (3, 6, 12, 24, and 48 h) and photographed under a phase-contrast microscope. Images taken at 20x were processed for quantification of number (**c**) and size (**d**) of vacuoles per single cell as in Materials and methods. **e** DLD-1 cells were transfected with either a specific siRNA for CK2α or a scrambled siRNA (control) by using Lipofectamine 2000 at a 1:3 ratio. Phase-contrast images were obtained at 0, 6, and 24 h post-transfection and quantification of size (gray bars) and number of vacuoles per single cell (black bars) were determined. Data represents the average ± SEM (*n* = 3); *p < 0.05; **p < 0.01; ***p < 0.001; ****p < 0.0001; ns: not significant. Bar: 20 μm

The onset of autophagy by ULK-1 activation normally leads to phagophore formation, which then fuses with lysosomes to form an autophagolysosome^13^. Interestingly, massive large cytosolic vacuoles formed only in CRC cell lines treated for 6 h with silmitasertib and not in normal CoN cells (Fig. 5a and Supp Fig. 4A). In DLD-1 cells, a progressive dose-dependent increase of small (<3 μm) vacuoles was observed with silmitasertib up to 12 h. At higher times, the number of vacuoles decreased concomitant with the increase in silmitasertib concentration while displaying an increase in their size, suggesting a fusion of small vacuoles to form bigger structures of almost 5–6 μm. At both higher silmitasertib concentrations and treatment times, a cellular collapse was evidenced by large birefringent structures, indicative of massive cell death (Fig. 5b–d). In addition, viability decreased in a time-dependent manner, showing significance after 12 h (Supp Fig. 4B). Interestingly, the number of small (<2 μm) vacuoles progressively increased up to 24 h, while significantly dropping at 48 h, which correlated with a significant increase of large (>4.5 μm) vacuoles. To confirm that the effect of silmitasertib on vacuole formation is caused by specific inhibition of CK2, expression of CK2α was silenced with the same siRNA as above (see Fig. 4b). Thus, CK2 silencing was sufficient to produce massive vacuole formation (≈4 μm), which progressed both in number and size in time, reaching larger structures (>5 μm) after 24 h of cell growth, resembling those triggered by silmitasertib at either 25 μM for 48 h or 50 μM for 24 h (Fig. 5e). As control, DLD-1 cells were treated with 100 ng/ml nocodazole for 24 h, which was unable to preclude the silmitasertib-dependent massive vacuolization and thus discarding that this process is product of a G2/M arrest (Supp Fig. 4C).

**Fig. 6:**
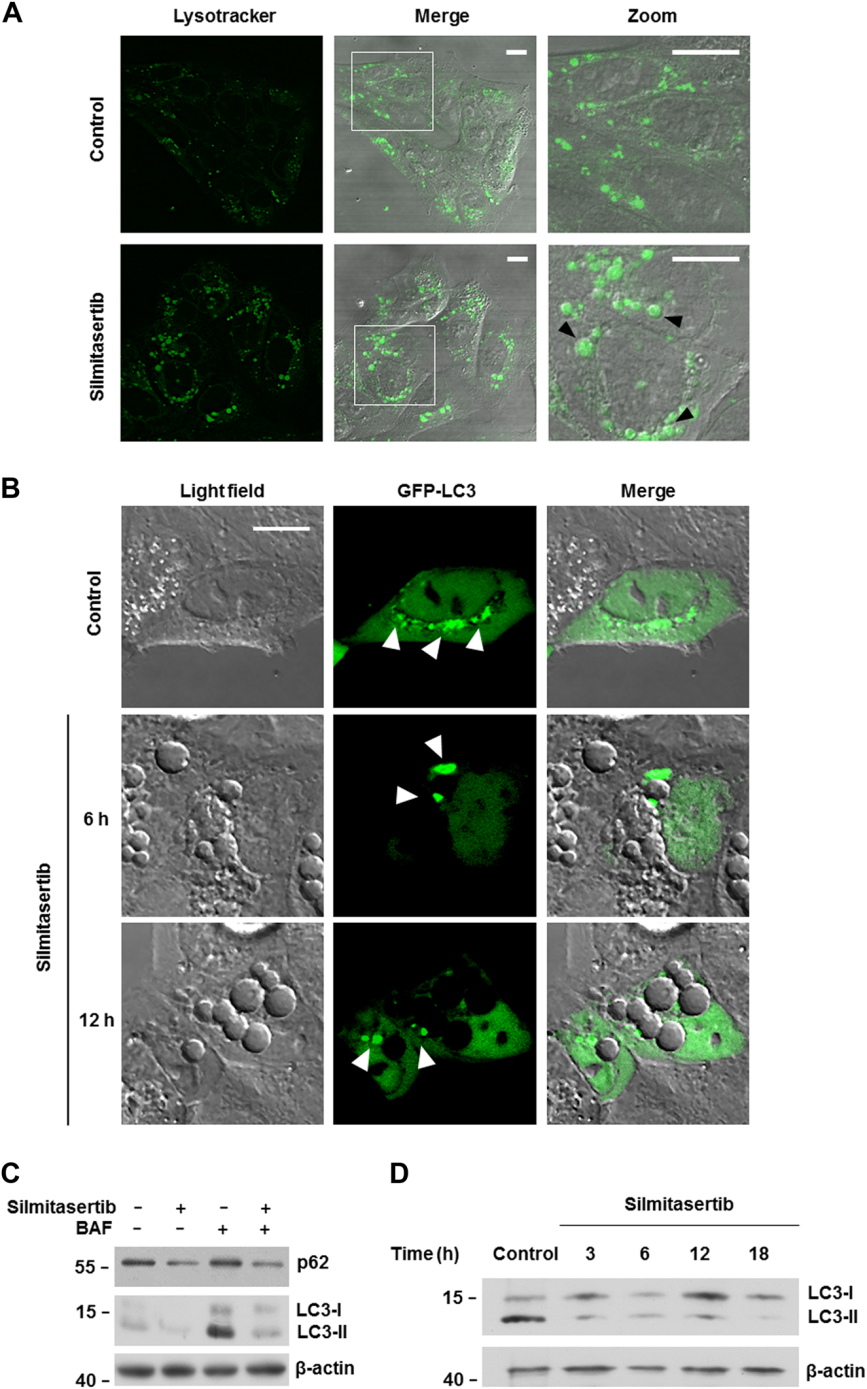
Silmitasertib-derived vacuoles are acidic but negative for LC3-II and p62. **a** DLD-1 cells treated with 25 μM silmitasertib for 6 h were then labeled with 1 μM lysotracker (green) for 30 min and living cells were visualized by video-microscopy. Black arrowheads indicate the vacuoles highlighted in white squares. Representative images are shown (*n* = 3), scale bars: 10 μm. **b** DLD-1 cells were transfected with a plasmid encoding GFP-LC3. After a 24 h transfection, cells were treated with 25 μM silmitasertib for 6 or 12 h and visualized by confocal microscopy. White arrowheads indicate LC3 puncta. **c** DLD-1 cells were treated during 6 h with 25 μM silmitasertib and/or 100 nM bafilomycin-A1 (BAF), followed by analysis of LC3-I/II and p62 levels by western blot. **d** DLD-1 cells were treated with 25 μM silmitasertib and the last 3 h with 100 nM BAF, then cultured for another 3, 6, 12, and 18 h, followed by analysis of LC3-I/II levels by western blot

To investigate whether those vacuoles correspond to autophagolysosomes, DLD-1 cells were loaded with lysotracker, which fluoresces in acidic pH like in lysosomes. As observed in Fig. 6a, vacuoles formed by treatment with silmitasertib were marked with lysotracker, indicating an acidic inner environment and which, due to their large size, could correspond to autophagolysosomes. During autophagy, ULK-1 phosphorylates and activates a class III PI3K, promoting the docking of the ubiquitin-like factor LC3-II to autophagolysosome membranes, which binds to its receptor p62 and together are ultimately degraded at the expanding autophagolysosome^13^. Thus, to assess whether silmitasertib-dependent ULK-1 activation and vacuolization are related to induction of autophagy, GFP-LC3 protein was expressed in DLD-1 cells and its subcellular distribution was evaluated after treatment with silmitasertib up to 12 h. Unexpectedly, untreated control cells showed GFP-tagged structures suggesting docking of LC3 at autophagolysosomes, which diminished after treatment for 6 h and almost completely after 12 h. Importantly, large vacuoles already present at 6 h did not recruit GFP-LC3 to their membranes at 12 h (Fig. 6b and Supp Fig. 5A). Indeed, similar results were obtained in HeLa cervical cancer cells, where large vacuoles formed under treatment with silmitasertib, which were also GFP-LC3-negative (Supp Fig. 5B). Additionally, decreased LC3-II and p62 levels were observed in DLD-1 cells treated with silmitasertib and bafilomycin-A1, an inhibitor of autophagic flow, for a period of up to 18 h (Fig. 6c, d). Importantly, this phenomenon was not related to *de novo* expression of representative autophagy genes, such as Beclin-1 and LC3 (Supp Fig. 5C) and vacuole formation was not blocked by inhibition of autophagy with 3-methyladenine (Supp Fig. 5D), inhibition of apoptosis with Z-VAD-FMK (Supp Fig. 5E), or inhibition of necroptosis with Nec1s (Supp Fig. 5F). Thus, our results suggest that silmitasertib-dependent mTORC1 inhibition, despite leading to the formation of acidic large LC3-negative vacuoles, they do not derive from induction of either autophagy, apoptosis or necroptosis.

### Vacuoles derive from a massive silmitasertib-dependent macropinocytosis

**Fig. 7:**
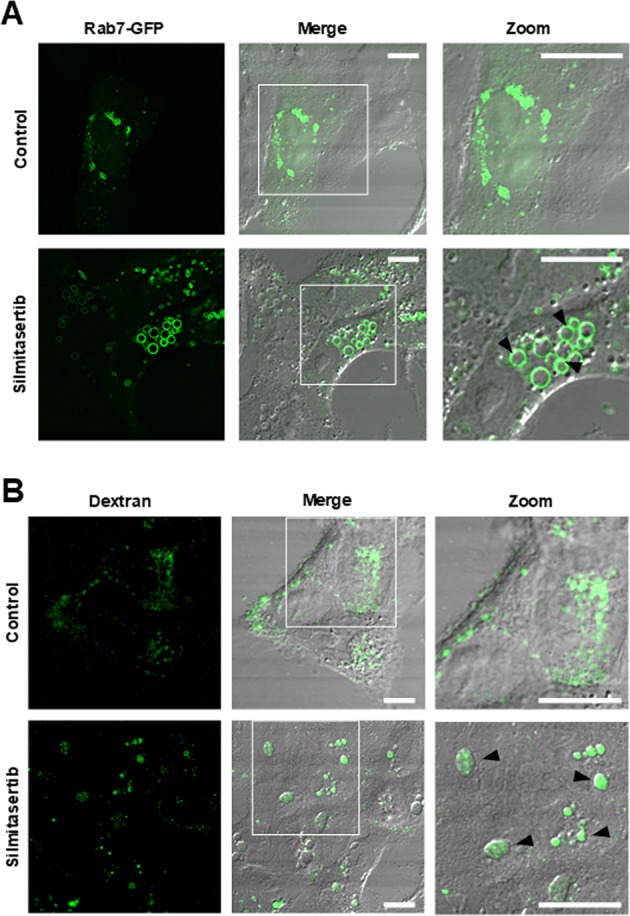
Macropinocytosis-like traits of silmitasertib-derived vacuoles in CRC cells. **a** DLD-1 cells were transfected with a plasmid encoding Rab7-GFP for 24 h and then treated with 25 μM silmitasertib for 6 h and finally visualized by confocal microscopy. **b** Cells were treated with silmitasertib as in **a**, then incubated for 1 h with 50 μg/ml FITC-dextran (green) and living cells were visualized by confocal microscopy. Black arrowheads indicate the vacuoles highlighted in white squares. Representative images are shown (*n* = 3); Bars: 10 μm

Since the large acidic vacuoles induced by silmitasertib do not have LC3 on their membranes, they could correspond to an endosomal compartment distinct from autophagolysosomes. Interestingly, we observed that silmitasertib-dependent vacuolization is precluded by bafilomycin-A1 treatment (Supp Fig. 5A), which has been shown to impair the macropinocytosis pathway elsewhere^40^. Moreover, the late endosomal marker Rab7 has been reported as an indicator of macropinosomes^41,42^. Indeed, early large vacuoles formed after treatment with silmitasertib were significantly Rab7 positive at 6 h (Fig. 7a). Similarly, another late endosomal marker, LAMP1, was also located at the membrane of such vacuoles after treatment with silmitasertib (Supp Fig. 6). Therefore, silmitasertib-promoted vacuoles in CRC cells correspond to an endolysosomal compartment similar to macropinosomes.

Because macropinocytosis is an endocytic pathway that mediates non-selective uptake of solutes, we used dextran to confirm the endocytic nature of the silmitasertib-derived vacuoles. An internalization assay was performed using 10 kDa FITC-conjugated dextran. Cells were treated with silmitasertib during 6 h and then incubated with dextran for 1 h. Consistently, control cells showed a constitutive level of endocytosis, however, the green fluorescence was concentrated at the large vacuoles observed following treatment with silmitasertib (Fig. 7b). Taken together, these results strongly suggest that inhibition of CK2 with silmitasertib leads to massive macropinocytosis in CRC cells.

## Discussion

Protein kinase CK2 is elevated in different cancers including CRC and its expression and activity are strongly linked to hyperproliferation and resistance to apoptosis, suggesting an important role in tumorigenesis^21^. In fact, CK2 has been proposed as a putative prognostic marker in cancer^43–45^. Consistently, an idea has emerged that some tumor cells become “CK2-addicted” in order to create a permissive microenvironment for tumor progression and to eventually favor an aggressive phenotype^23^. Thus, inhibition of CK2 should be extremely catastrophic in those cancer cells. Therefore, CK2 has evolved as an attractive therapeutic target in cancer, with two pharmacological inhibitors extensively used in in vitro and ex vivo experiments, TBB (4,5,6,7-tetrabromobenzotriazole) and silmitasertib (formerly CX-4945), the latter having been tested in clinical trials. Both are CK2 competitive inhibitors for the ATP binding site, normally used at the micromolar scale by us and other groups^46,47^. However, silmitasertib had a more potent effect than TBB in decreasing viability of CRC cells, since a four-fold lower concentration of the former was used in this work to achieve the same effect (data not shown). Indeed, DLD-1 cells showed significant decrease in in vitro tumorigenesis after 21 days, accounting for an extremely deleterious effect of silmitasertib on cell viability. This correlated with early cell cycle arrest at G2/M, previously seen in breast, lung and glioblastoma cancer cells^27,48,49^. Of note, the silmitasertib-dependent arrest in our DLD-1 cells was distinct to what we observed in HT-29 cells treated with TBB for 24 h^35^. Also relevant, the G2/M arrest was irrespective of activating mutations in the *PIK3CA* gene, a common trait found in CRC tumors^2^, since the same was observed in SW-480 cells, which are free of the genomic alteration. This suggests a specific and broad effect of silmitasertib on any CRC cell, endorsing its use in CRC patient therapy in the future.

Our data show that CK2 may activate the PI3K/Akt/mTORC1 signaling pathway in CRC. It has been described that silmitasertib inhibits this pathway and hence decreases viability of lung cancer cells^27^. Additionally, silmitasertib in combination with erlotinib (an EGFR inhibitor) produces a complete inhibition of this pathway, inducing apoptosis in squamous lung cancer cells^28^. Moreover, two pharmacological inhibitors of mTORC1, everolimus and temsirolimus, exemplify the therapeutic importance of the inhibition of this pathway in patients with advanced renal carcinoma^50^. Thus, taking into consideration our published findings on the functional relationship between CK2 and Akt^36,37^, an attractive possibility emerged where CK2 may eventually upregulate mTORC1 via phosphorylation/activation of Akt in CRC cells. However, overexpression of the CK2-phosphorylation resistant mutant, Akt-S129A, behaved similarly to wild-type Akt on mTORC1 activity. This could be a consequence of an mTORC1-independent effect since activation of mTORC1 by Akt involves different proteins. For example, CK2 may alter the activity of the Rags proteins, which are responsible for mTORC1 anchorage at the lysosome membrane^51^.

Silmitasertib-dependent inhibition of mTORC1 has been seen to promote autophagy in lung, pancreas and glioblastoma cancer cells, where inhibition of CK2 led to inhibition of mTORC1 and displayed features of autophagy^26,27,52^. Interestingly, it has also been described that a decrease in the expression and activity of CK2 leads to the formation of large vacuoles in pancreatic cancer cells, related to decreased viability and autophagy induction^26^. Consistent with this, we observed the formation of early large acidic vacuoles in DLD-1 cells treated with silmitasertib. In fact, these vacuoles had been previously seen in CRC cells following incubation with other less-potent CK2 inhibitors, including apigenin and DRB, with no explanation about their origin (Tapia J.C., personal communication). These vacuoles lacked LC3 on their membranes and the LC3-II and p62 levels did not correlate with early autophagy induction^53^. Instead, silmitasertib inhibited accumulation of LC3-II and p62 in presence of bafilomycin-A1, indicative of autophagy inhibition. This result is contradictory, considering that inhibition of CK2 with silmitasertib-promoted ULK-1 activation associated to the inhibition of mTORC1. Although it is not possible to rule out that the large vacuoles induced by silmitasertib somehow mask an induction of autophagy, CK2 may have a regulatory effect (e.g., by phosphorylation) on some key autophagy regulators, leading finally to an attenuation of autophagy induction after mTORC1 activation. In fact, p62 is phosphorylated by CK2 at Ser-403, thereby favoring autophagic clearance of ubiquitinated targets^54^. Likewise, ULK-1 contains putative phosphorylation sites for CK2, one (Thr-109) located in its kinase domain, although if the site(s) are functionally relevant for autophagy is yet unknown.

Silmitasertib is a well-known inducer of cell death in different cancer cells, including CRC, although the underlying mechanism not only involves apoptosis but also other non-apoptotic mechanisms. The difficulty for precisely determine the death mechanism is probably due to the multiple targets that CK2 phosphorylates involved in several cancer-related processes^23^. Here, we determined that the viability of CRC cells treated with this inhibitor decreased 60–70%, of which only a 20% correspond to apoptosis at 48 h. However, autophagy and apoptosis are related cellular mechanisms. When autophagy is deficient or inhibited, apoptosis is induced, and conversely autophagy is induced upon deficient apoptosis^15^. This cross-regulation responds to the interaction of two regulatory proteins, Beclin-1 (autophagy inducer) and Bcl-2 (anti-apoptotic)^55^, which leads to the inhibition of autophagy-associated death. Although interesting, this mechanistic relationship was not addressed in this work and the observed cell death remains of unknown origin.

**Fig. 8:**
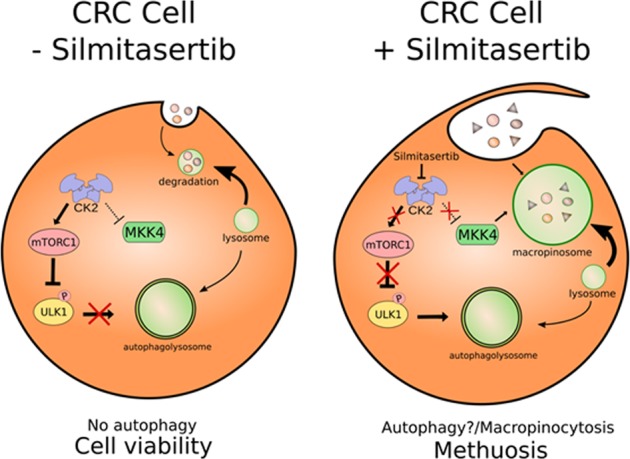
Pivotal role of CK2 in life and death of CRC cells by regulation of methuosis. In untreated cells (left) an aberrantly elevated expression/activity of CK2 promotes upregulation of mTORC1 and hence downregulation of ULK-1, promoting autophagy-independent cell viability. Here, MKK4 may also be downregulated by CK2, thereby not affecting macropinocytosis. In silmitasertib-treated cells (right), however, a putative MKK4-dependent macropinocytosis is promoted, where early massive formation of acidic vacuoles (LC3-II^−^/LAMP1^+^/Rab7^+^) then triggers a G2/M arrest and ultimately methuosis-like cell death. Consequently, CRC cells are unable to form tumors

Our findings indicate that silmitasertib induces a deep change in the endocytic pathway of CRC cells, where large acidic LC3-II^−^/LAMP1^+^/Rab7^+^ macropinosomes are massively formed (see Fig. 8). Indeed, recent data in literature support this mechanism, since some antineoplastic drugs promote massive vacuolization that eventually leads to necrotic-like cell death, often named methuosis^42,56–58^. Interestingly, the mitogen-activated protein kinase kinase 4 (MKK4) has been indicated as a key factor in macropinocytosis activation^58^ and its downregulation is associated with liver metastasis in CRC^59^. Moreover, silencing of the CK2α subunit leads to MKK4 activation and, consequently, significant death of PANC-1 human pancreatic cancer cells treated with gemcitabine^60^, which displays methuosis-like traits. Nevertheless, whether the putative CK2-dependent regulation of MKK4 is involved in CRC cell death as well as decreased in vitro tumorigenesis after 21 days, is an issue that warrants further research. Altogether, our findings point to this selective inhibitor of CK2 as a promissory therapeutic drug for CRC patients and probably other CK2-addicted cancers.

## Materials and methods

### Cell culture

Human normal colon cells CCD 841 (CoN) and colon cancer cell lines (SW-480, DLD-1, HT-29 and HCT-116) were purchased from ATCC. Cells were maintained at 37 °C and 5% CO_2_ in RPMI-1640 medium (Invitrogen, Paisley, UK) supplemented with 10% Fetal Bovine Serum (HyClone, Logan, UT), antibiotics (10000 U/ml penicillin and 10 μg/ml streptomycin) and eventually treated with silmitasertib (ApexBio Technology LLC, Houston, TX).

### Expression vectors and transfection

The pCMV6-myr-AKT-1-CA-HA plasmid^61^ was purchased from Addgene (Cambridge, USA) and pCMV6-myr-AKT-1-S129A-HA was a kind gift from Dr. Maria Ruzzene^25^. Expression vectors for Rab7-GFP, LAMP1-RFP, GFP-LC3, HA-CK2α and GFP-CK2α have been described elsewhere^17,62^. Small interfering RNAs (siRNA) targeted to CK2α and control siRNA were purchased from Santa Cruz Biotechnology (Santa Cruz, CA). Transfections were carried out with 2 μg each DNA by using Lipofectamine 2000 as reagent, according to the manufacturer’s instructions (Life Technologies, Rockford, IL).

### Western blot

Cells were lysed in RIPA buffer (Thermo Scientific, Rockford, IL) with the cocktail of protease and phosphatase inhibitors PhoSTOP (Roche, Mannheim, Germany). Proteins were separated by sodium dodecyl sulfate polyacrylamide gel electrophoresis, transferred to NitroPure membranes (Macherey-Nagel, Dϋren, Germany) and blocked with 5% BSA (Thermo Scientific, Rockford, IL) in PBS/0.1% Tween. Blots were incubated with primary antibodies (Cell Signaling Technology, Beverly, MA): rabbit polyclonal anti-PARP (1:1000), rabbit polyclonal anti-CK2α (1:1000), rabbit polyclonal anti-LC3 (1:1000), rabbit monoclonal anti-pSer757-ULK-1 (1:1000), rabbit monoclonal anti-ULK-1 (1:1000), rabbit monoclonal anti-Akt (1:1000), rabbit monoclonal anti-pThr389-S6K1 (1:1000), rabbit polyclonal anti-S6K1 (1:1000), rabbit polyclonal anti-pSer235/Ser236-S6 (1:1000) and mouse monoclonal anti-S6 (1:1000). Mouse monoclonal anti-p62 (1:1000) was obtained from BD Biosciences and anti-actin (1:2000) from Santa Cruz Biotechnology (Santa Cruz, CA). The secondary antibodies used were: anti-goat IgG-HRP (1:2000), anti-rabbit IgG-HRP (1:2000) and anti-mouse IgG-HRP (1:2000) from Santa Cruz Biotechnology. Blots were revealed with the EZ-ECL chemiluminescent kit (Biological Industries, Haemek, Israel).

### Immunofluorescence and confocal microscopy

Cells were grown on glass coverslips for the indicated times, washed with PBS, fixed in 3.7% p-formaldehyde for 15 min, permeabilized for 10 min at room temperature (RT) in 0.2% Triton X-100/PBS (i.e., PBST), blocked with 3% BSA/PBST for 30 min at RT and incubated with primary antibody in 1% BSA/PBST (1:200 for anti-mTOR and 1:200 for anti-HMGB1) for 1 h at RT. Incubation with secondary antibody (1:500) was performed in 1% BSA/PBST for 45 min at RT in the dark. Lysosomes were stained with 1 μM lysotracker for 30 min and nuclei with DAPI for 10 min (Thermo-Fisher Scientific). For dextran uptake, cells were incubated for 1 h with 50 μg/ml FITC-conjugated dextran (Thermo-Fisher Scientific). For some experiments, slides were mounted in ProLong® (Life Technologies) and images obtained with an Olympus-IX81 DSU Spinning Disk confocal microscope. For video-microscopy experiments, cells seeded on glass coverslips were mounted on a microscope chamber equipped with a heating unit (37 °C) and 5% CO_2_, and then analyzed by fluorescence microscopy using an Olympus Confocal FV1000. The program FV10-ASW 3.0 was used for all acquisitions and settings. For determination of number and size of vacuoles, images were taken at 20x magnification, segmented and binarized on the ImageJ software by homemade commands, and put all together in a macro file. By using the /Window sincronization function on ImageJ, circular regions corresponding to vacuoles were tracked on binary images by contrasting them with the original ones. Size (diameter) and number of vacuoles identified were automatically measured by the /Multi measure function on ImageJ.

### Viability

Cells growing at 50–60% confluency on six-well plates were transfected and/or treated with inhibitor as indicated in the figure, left in culture during 24 h and subsequently re-plated in 96-well plates at a density of 1 × 10^4^ cells per well. Viability was measured using the MTS® assay according to the manufacturer’s instructions (Promega).

### Flow cytometry

For proliferation assay, cells were detached by trypsinization, washed and left in PBS to a density of 10^6 ^cells/ml. CFSE (eBioscience) was added to a final concentration of 5 μM, mixed for 10 s and incubated for 10 min. One volume of FBS was added, followed by complete medium and cells were then pelleted, washed, seeded into p60 petri dishes, and incubated with DMSO (dimethyl sulfoxide) or silmitasertib for 48 h. For apoptosis assay, cells (2 × 10^5^) were washed with PBS and left in a total volume of 100 μl binding buffer (2.5 mM CaCl_2_, 0.01 M Hepes, 0.14 M NaCl). One microliter annexin-V-FITC (Biolegend) and 2 μl 50 μg/ml propidium iodide (PI) were added and cells incubated for 15 min at RT. Finally, 300 μl binding buffer was added to stop the reaction. For cell cycle assay, cells were incubated with 25 μM silmitasertib for the indicated times and then centrifuged at 1000 x *g* for 5 min at RT, washed in 1 ml cold PBS, suspended in 1 ml staining solution containing 0.1% Triton X-100, 50 μg/ml PI and 200 μg/ml RNAse, and incubated for 30 min at 37 °C in the dark. All assays were analyzed on a Becton-Dickinson LSR Fortessa X-20 flow cytometer and the FACSDiva 8.02 software (San Jose, CA) at the MED.UCHILE-FACS Facility, Facultad de Medicina, Universidad de Chile.

### Anchorage-independent growth

Cells (2.5 × 10^3^) were suspended in 0.33% Bacto-agar (BD Biosciences) in a media containing 12.5% FBS in RPMI-1640. The cell suspension was then poured into 6-well plates containing a layer of 2 ml 0.5% agar in the same media. Plates were fed twice a week with 0.5 ml RPMI-1640 supplemented with 10% FBS. After 21 days, colonies were photographed under a Nikon Eclipse TS100 inverted microscope. Finally, cells were stained using 0.005% crystal violet dissolved in 20% methanol for 1 h at RT and colonies visible to the naked eye were also photographed and documented with a Nikon D5100 camera.

### Migration

Migration assay was performed in Boyden Chambers of 6.5 mm diameter and 8 μm pore, according to manufacturer’s instructions (Transwell Costar). Briefly, the bottom of each insert was coated with 0.5 ml 2 μg/ml fibronectin. Cells (3.5 × 10^4^) were suspended in serum-free medium and seeded onto the top of each chamber insert. Medium supplemented with 10% FBS was added to the bottom chamber. After 8 h, inserts were removed, washed, and cells adhered to the bottom side of the inserts were stained with 0.1% crystal violet in 20% methanol. The number of migrated cells was determined under a Nikon Eclipse TS100 inverted microscope.

### Reverse transcription quantitative PCR (RT-qPCR)

Total RNA from cells was extracted by using the EZNA Total RNA Kit I (Omega bio-tek, Giorgia USA) and treated with DNase (DNA-free Kit Ambion, Life Technologies). RNA concentration was measured with NanoQuant Infinite M200 pro spectrophotometer (Tecan). Reverse transcription was performed by using the AffinityScript QPCR cDNA Synthesis Kit (Agilent Technologies, TX). Quantitative real-time PCR was performed in a StepOne real-time PCR system (Applied Biosystems) with SYBR Green PCR master mix (Thermo-Fisher Scientific, Vilnius, Lithuania). Determinations were performed in triplicate (40 cycles) and the relative abundance of each mRNA was determined by using the 2^-ΔΔct^ method and normalized with GAPDH. The limits of a 95% confidence interval were determined to indicate variability of the mean ratios for each experiment. Primers used were: Beclin-1 Fw 5´-ACCGTGTCACCATCCAGGAA-3´ and Rv 5´-GAAGCTGTTGGCACTTTCTGT-3´. LC3 Fw 5´-CCGTCGGAGAAGACCTTCAA-3’ and Rv 5´-GCATAGACCATGTACAGGAA-3´; GAPDH Fw 5´-GAGTCAACGGATTTGGTCGT-3’ and Rv 5´-GACAAGCTTCCCGTTCTCAG-3´.

### Statistics

All values were expressed as mean ± SEM of three independent experiments. Data was analyzed using two-tailed unpaired Student’s *t*-test and ANOVA. *p*-value was set at a nominal level of 0.05 or less.

## Supplementary information

Supplementary Figure 1

Supplementary Figure 2

Supplementary Figure 3

Supplementary Figure 4

Supplementary Figure 5–1

Supplementary Figure 5–2

Supplementary Figure 5–3

Supplementary Figure 6

Supplementary figure legends
