# Supplementary figures and images for: CK2 inhibition with silmitasertib promotes methuosis-like cell death associated to catastrophic massive vacuolization of colorectal cancer cells
Source: Cell Death Dis. 2019 Jan 25;10(2):73. doi: 10.1038/s41419-019-1306-x (PMC6347595; doi:10.1038/s41419-019-1306-x)

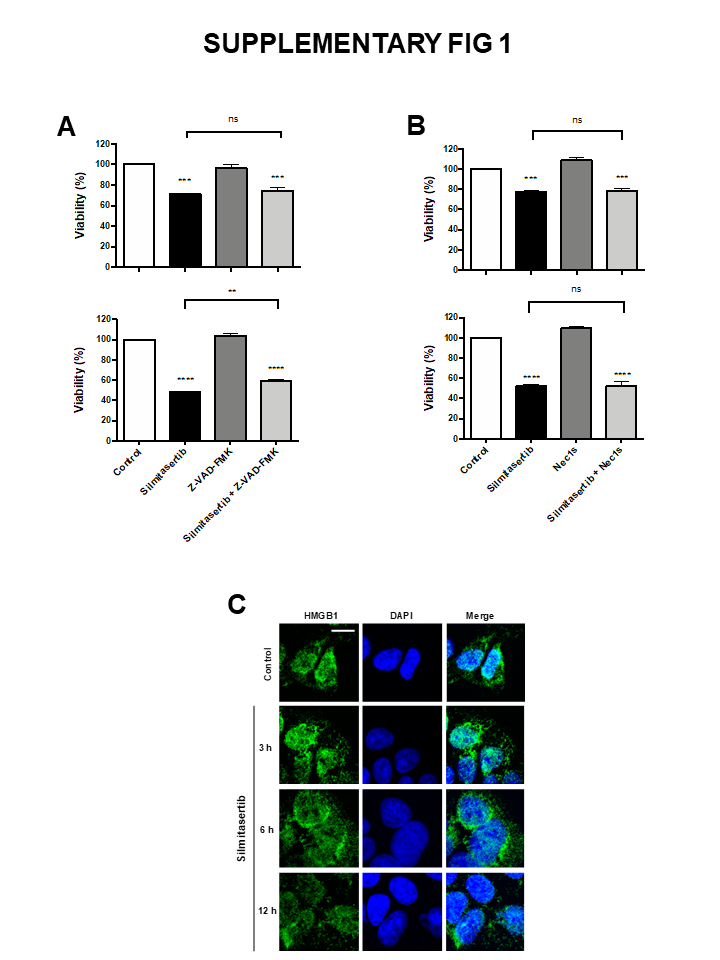

Supplement: Supplementary file 1 — Supplementary Figure 1 [file 41419_2019_1306_MOESM1_ESM.tif]

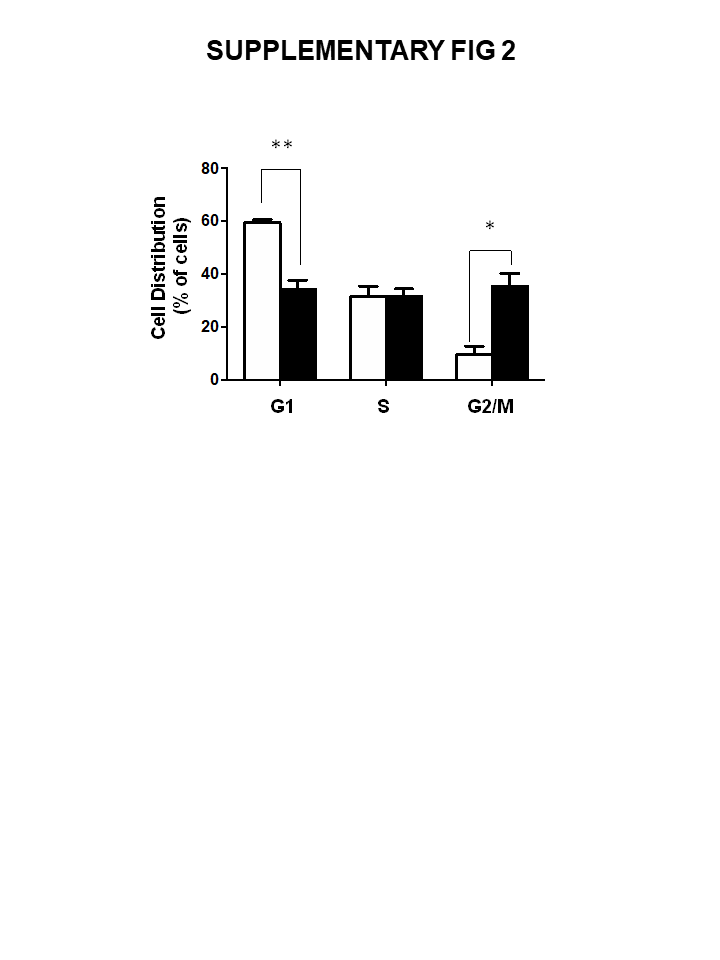

Supplement: Supplementary file 2 — Supplementary Figure 2 [file 41419_2019_1306_MOESM2_ESM.tif]

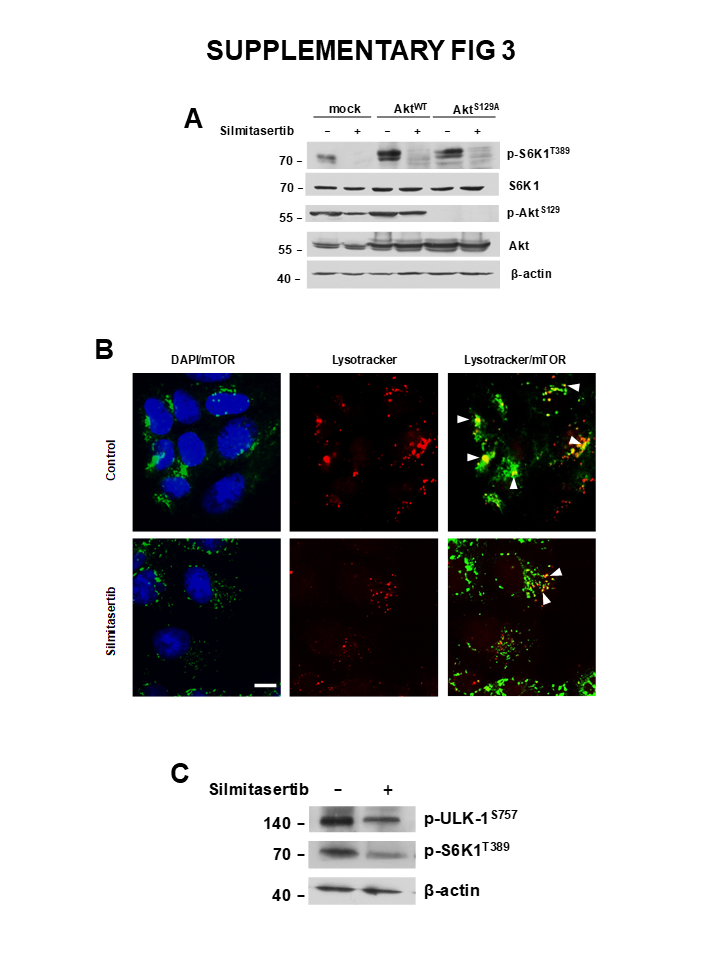

Supplement: Supplementary file 3 — Supplementary Figure 3 [file 41419_2019_1306_MOESM3_ESM.tif]

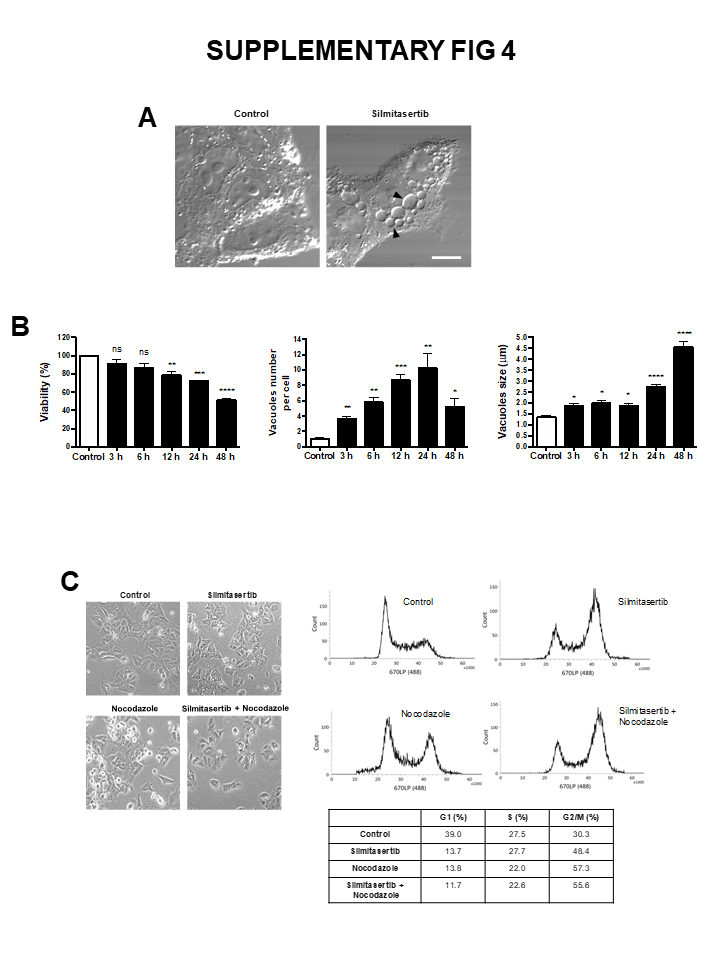

Supplement: Supplementary file 4 — Supplementary Figure 4 [file 41419_2019_1306_MOESM4_ESM.tif]

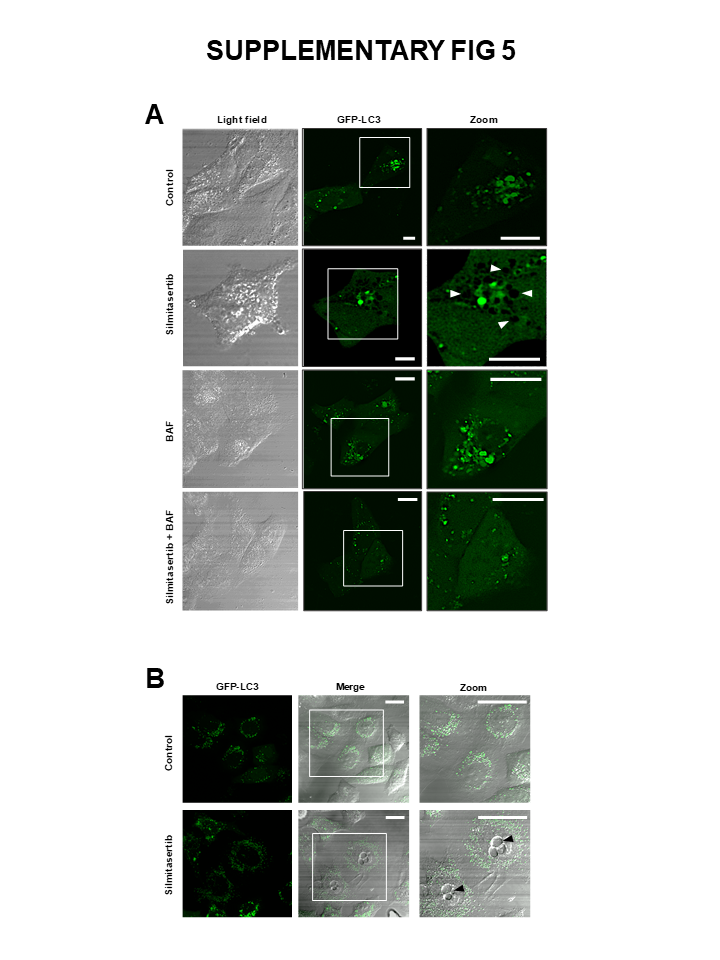

Supplement: Supplementary file 5 — Supplementary Figure 5–1 [file 41419_2019_1306_MOESM5_ESM.tif]

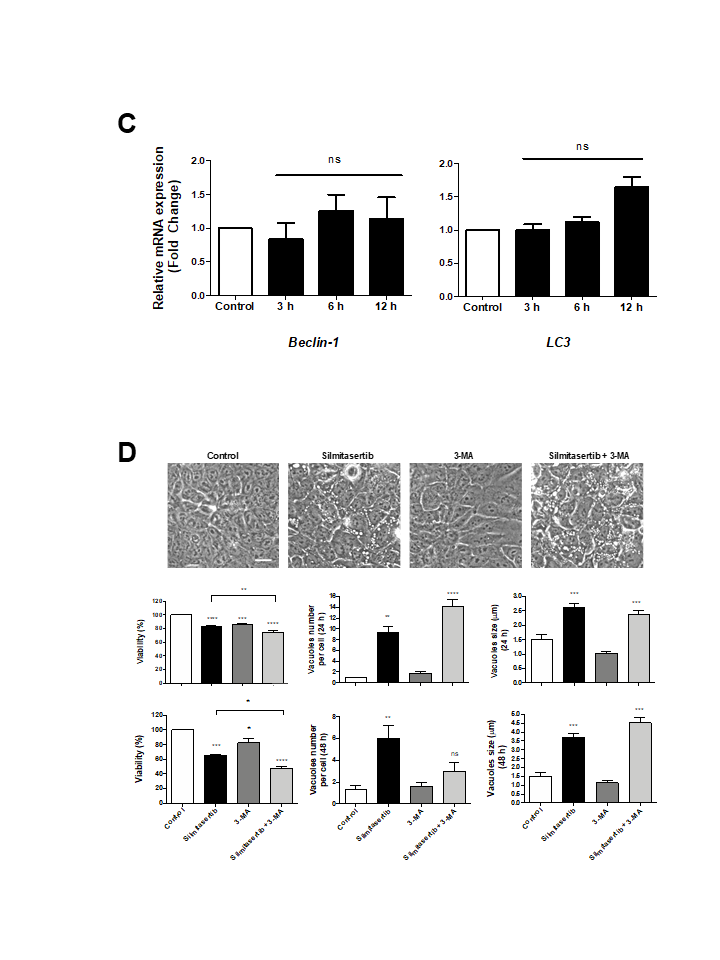

Supplement: Supplementary file 6 — Supplementary Figure 5–2 [file 41419_2019_1306_MOESM6_ESM.tif]

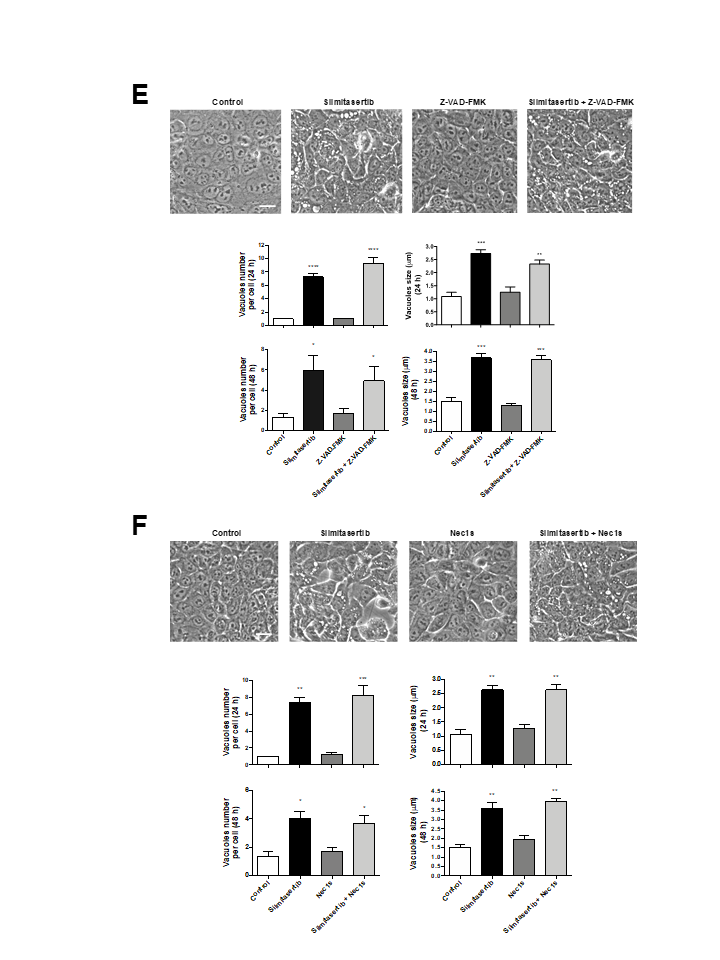

Supplement: Supplementary file 7 — Supplementary Figure 5–3 [file 41419_2019_1306_MOESM7_ESM.tif]

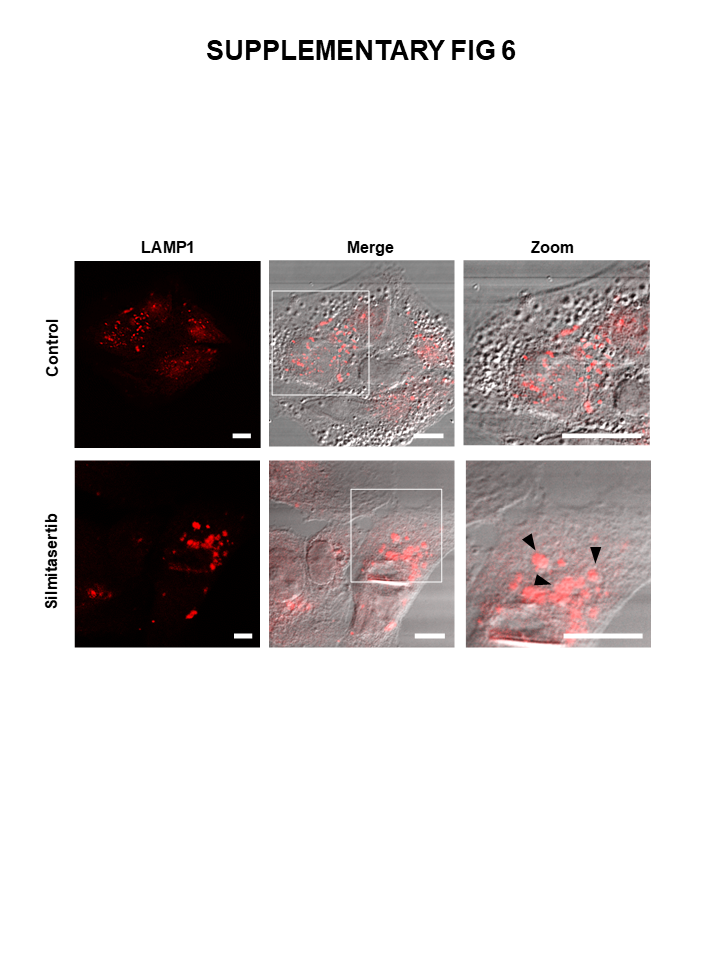

Supplement: Supplementary file 8 — Supplementary Figure 6 [file 41419_2019_1306_MOESM8_ESM.tif]
