# Supplementary material for: CK2 inhibition with silmitasertib promotes methuosis-like cell death associated to catastrophic massive vacuolization of colorectal cancer cells
Source: Cell Death Dis. 2019 Jan 25;10(2):73. doi: 10.1038/s41419-019-1306-x (PMC6347595; doi:10.1038/s41419-019-1306-x)
Supplement: Supplementary file 9 — Supplementary figure legends [file 41419_2019_1306_MOESM9_ESM.docx]

**SUPPLEMENTARY FIGURE LEGENDS**

**Supplementary Figure 1. Massive vacuolization of CRC cells does not relate with necrosis or necroptosis. (A)** Cell viability was determined by MTS assay in DLD-1 cells after incubation with 25 μM silmitasertib with or without the pan-caspase inhibitor 30 μM Z-VAD-FMK for 24 h (upper) and 48 h (lower). **(B)** Cell viability determined by MTS after incubation with 25 μM silmitasertib with or without necroptosis inhibitor 30 μM Nec1s for 24 h (upper) and 48 h (lower). **(C)** DLD-1 cells were treated with 25 μM silmitasertib for 3, 6 and 12 h and then observed by confocal microscopy using a specific antibody for HMGB1 and DAPI for nuclei. A representative image is shown. Bar: 10 μm.

**Supplementary Figure 2. Silmitasertib promotes G2/M arrest in SW-480 cells.** Cell cycle distribution was determined by flow cytometry of PI-stained CRC cells treated with 25 μM silmitasertib for 24 h. The percentage of cells in the different cell cycle phases is plotted. Data represents the average ± SEM (n=3); *p < 0.05, **p < 0.001.

**Supplementary Figure 3. Silmitasertib hinders activation of mTORC1 in CRC cells.** **(A)** DLD-1 cells transfected with the plasmids pCMV6-myr-AKT-1-CA-HA or pCMV6-myr-AKT-1-S129A-HA. After 24 h, cells were treated with 25 μM silmitasertib for 6 h. The levels of p-S6K1^T389^, S6K1, p-Akt^S129^, Akt and β-actin were evaluated by Western blot with specific antibodies. **(B)** DLD-1 cells were treated with 25 μM silmitasertib for 6 h and then observed by confocal microscopy using a specific antibody for mTOR, lysotracker for lysosomes and DAPI for nuclei. White arrowheads indicate mTORC1 co-localizating with lysosomes (yellow). A representative image is shown. Bar: 10 μm. **(C)** DLD-1 cells were treated for 6 h with 25 μM silmitasertib, then levels of pSer757-ULK-1 and pThr389-S6K-1 were evaluated by Western blot. A representative figure of 3 independent experiments is shown.

**Supplementary Figure 4. Silmitasertib induces massive formation of large vacuoles in CRC cells. (A)** DLD-1 cells were treated with 25 μM silmitasertib for 6 h and visualized by Nomarski microscopy. Black arrowheads indicate the cytoplasmic vacuoles. A representative image is shown. Bar: 10 μm. **(B)** Cell viability was determined by MTS assay in DLD-1 cells treated with 25 μM silmitasertib for 3, 6, 12, 24 and 48 h. The number and size of vacuoles were plotted. **(C)** DLD-1 cells were treated with 25 μM silmitasertib and/or 100 ng/ml nocodazole for 24 h, then visualized by microscopy and analyzed by FACS with PI staining to evaluate cell cycle arrest. Data represent the average ± SEM (n=3). ﻿*p ≤ 0.05; ﻿**p ≤ 0.01; ﻿***p ≤ 0.001; ﻿****p ≤ 0.0001; ns, not significant.

**Supplementary Figure 5. Silmitasertib-induced vacuoles are LC3-II negative and refractory to inhibitors of cell cycle, apoptosis and autophagy. (A)** DLD-1 cells were transfected with a plasmid encoding GFP-LC3. After 24 h, cells were treated during 6 h with 25 μM silmitasertib and the last 3 h with 100 nM bafilomycin-A1 (BAF) and visualized by confocal microscopy. White arrowheads indicate the vacuoles highlighted in the white square. A representative image is shown. Bars: 10 μm. **(B)** Human cervical HeLa cancer cells stably overexpressing GFP-LC3 were treated with 25 μM silmitasertib for 6 h and then visualized by confocal microscopy. Black arrowheads indicate the vacuoles highlighted in white squares. A representative image is shown. Bars: 10 μm. **(C)** DLD-1 cells were treated with 25 µm silmitasertib for 3, 6 and 12 h and after purifying total RNA the relative Beclin-1 and LC3 mRNA levels were measured by RT-qPCR. **(D)** DLD-1 cells after incubation with 25 μM silmitasertib with or without autophagy inhibitor 5 mM 3-methyladenine (3-MA) were visualized by phase-contrast microscopy (upper). Cell viability, number and size of vacuoles were plotted after 24 h (middle) and 48 h (lower) of treatment. Bars: 20 μm. **(E)** DLD-1 cells were treated with 25 μM silmitasertib with or without pan-caspase inhibitor 30 μM Z-VAD-FMK and visualized by phase-contrast microscopy (upper). Number and size of vacuoles were plotted after 24 h (middle) and 48 h (lower) of treatment. Bars: 20 μm. **(F)** DLD-1 cells were treated with 25 μM silmitasertib with or without necroptosis inhibitor 30 μM necrostatine-1s (Nec1s) and visualized by phase-contrast microscopy (upper). Number and size of vacuoles were plotted after 24 h (middle) and 48 h (lower) of treatment. Bars: 20 μm. Data represent the average ± SEM (n=3); ﻿*p ≤ 0.05; ﻿**p ≤ 0.01; ﻿***p ≤ 0.001; ﻿****p ≤ 0.0001; ns, not significant.

**Supplementary Figure 6. Silmitasertib-derived vacuoles are LAMP1 positive in CRC cells.** DLD-1 cells were transfected with a plasmid encoding LAMP1-RFP. After 24 h, cell were treated with 25 μM silmitasertib for 6 h, and visualized by confocal microscopy. Black arrowheads indicate the vacuoles highlighted in white squares. A representative image is shown (n=3); Bars: 10 μm.
